# Competing Logics and Healthcare: Comment on "(Re) Making the Procrustean Bed? Standardization and Customization as Competing Logics in Healthcare"

**DOI:** 10.15171/ijhpm.2017.100

**Published:** 2017-08-20

**Authors:** Mike Saks

**Affiliations:** ^1^University of Suffolk, Ipswich, UK.; ^2^University of Lincoln, Lincoln, UK.; ^3^Royal Veterinary College, University of London, London, UK.; ^4^University of St Mark and St John, Plymouth, UK.; ^5^University of Toronto, Toronto, ON, Canada.

**Keywords:** Standardization, Customization, Institutional Logics, Healthcare, Complementary and Alternative Medicine (CAM)

## Abstract

This paper offers a short commentary on the editorial by Mannion and Exworthy. The paper highlights the positive insights offered by their analysis into the tensions between the competing institutional logics of standardization and customization in healthcare, in part manifested in the conflict between managers and professionals, and endorses the plea of the authors for further research in this field. However, the editorial is criticized for its lack of a strong societal reference point, the comparative absence of focus on hybridization, and its failure to highlight structural factors impinging on the opposing logics in a broader neo-institutional framework. With reference to the Procrustean metaphor, it is argued that greater stress should be placed on the healthcare user in future health policy. Finally, the case of complementary and alternative medicine is set out which – while not explicitly mentioned in the editorial – most effectively concretizes the tensions at the heart of this analysis of healthcare.

## Introduction: Applying Competing Logics to Healthcare

The editorial by Russell Mannion and Mark Exworthy on ‘(Re) Making a Procrustean Bed? Standardization and Customization as Competing Logics in Healthcare’ is both well written and well observed.^1^ Drawing on more general organizational theory in developing its approach, it accentuates the tension between the opposing institutional logics of standardization and customization in the health arena specifically. On the positive side, the editorial is usefully based on the design, technological, performance and procedural standards that underpin the standardization process – highlighting its links to the evidence-based medicine (EBM) movement. It also helpfully explores the criticisms that have been made of the EBM approach including its sometimes shaky ‘evidence’ base and the reliance on tacit knowledge – as well as the implications for healthcare delivery, from the tightening up of clinical governance procedures to clinical protocols relating to practice.

This is importantly noted to be manifested in the conflicting views of managers and professional clinicians, the latter of whom tend to value their own autonomy and adopt a more pragmatic approach in working with institutional rules. This in turn shades into the tension with customization which encompasses personalization and is based, amongst other things, on more individualized clinical intervention and partnerships with clients in healthcare, including co-production with ‘expert’ patients assuming a more active role in their own care. The authors are also right to suggest that there is a need for more work on how these opposing logics interface and co-exist – and are articulated, adapted and resisted – on the fault line that they create in many health systems. Here they may sometimes blend as well as decouple in relation to standardization/customization, especially at the meso and micro level, in a wider world frequently pervaded by complex multiple institutional logics – which may also encompass at a macro level, amongst others, democratic, professional, managerial and market logics.^2^ Either way, the institutional logics approach adds another dimension to insights provided by more traditional analyses of health policy focused simply on professions, bureaucracy and consumers.^3^

Against this, this editorial argument itself has fault lines. One of these is that the narrative implicitly focuses on the National Health Service in England without considering more specifically to which countries their analysis applies, apart from a loose reference to neo-liberal societies. Clearly it is relevant to numerous modern societies, but they do not examine whether and why variations exist between countries, given the different socio-political backgrounds of particular societies – based not just on cultural factors, but also the spectrum of arrangements from privatized to state-dominated public structures that can bring differing imperatives to the fore.^4^ Nor do they comment on how far the model of competing logics fits developing societies more globally – many of which are based on more traditional systems of healthcare, with sometimes very separate post-colonial dynamics?^5^ In part as a result of the interesting issues that this throws up, we shall explore further the question of complementary and alternative medicine (CAM) as a distinctive part of modern healthcare in the penultimate section of this commentary. The therapies concerned, whose maintenance and growth is currently encouraged as traditional medicine by the World Health Organization (WHO),^6^ frequently have roots in pre-industrial holistic practices and even today typically prioritize personalization over standardization.

Another fault line is that some of the better researched aspects of the tension in institutional logics are very lightly skated over in the account presented – especially that of hybridization which has recently been quite fully explored both generally in terms of typologies^7^ and more specifically in terms of the dilemmas that it poses, and how these are resolved, in different areas of healthcare like nursing^8^ in Western Europe and beyond. There is also no reference to some of the broader structural factors both nationally and internationally which impinge on the adoption of particular patterns of healthcare as regards personalization/standardization – including the impact of consumer lobbies, economic manoeuvring by pharmaceutical companies and the political stance of the state.^9^ This clearly leads further into the neo-institutional approach at a wider level in which professions and organizations are seen as voraciously competing for survival in an ecology of institutional forms, with all the consequent effects.^10^

## Valuing Users in Healthcare

Moving on, metaphors are very powerful in illuminating issues – be it in healthcare or, indeed, in other professional fields.^11^ The reference to ancient Greek mythology is therefore very apt in this context given the implicit attack on the ‘one size fits all’ conformity of standardization outlined in the editorial, which is represented by the Procrustean bed. However, the metaphor was not systematically followed through in relation to users who are sadly largely ignored in this analysis of this institutional logic. In particular, it might have been asked whether the unfortunate guests of Procrustes, the inn-keeper, could also be seen symbolically as consumers of health services, some of whose lives have been sacrificed from the standpoint of quality of life and longevity to the managerial idolatry of standardization. This is an important comment as the resolution of the tension between standardization and customization is not just a ‘compelling’ academic issue, but is critical to the wellbeing of users. The implications for clients of healthcare providers, though, do not significantly figure in the editorial – nor does the related need to provide the analytical tools to make a substantive judgement on the relative costs and benefits of the two logics from the viewpoint of consumers and the relevant balance to be struck between them. At a wider level, another metaphor drawing on ancient Greek mythology might be employed about the dangers to health systems of an overly standardized approach – that of Icarus with his wings of wax flying too close to the sun. Melt down clearly needs to be avoided from the viewpoint of users in striking an appropriate equilibrium.

As such, the analysis is a reminder of the classic work by Ivan Illich at the height of the 1960s/1970s counter culture, entitled *Limits to Medicine*.^12^ Here he explored another not too dissimilar fault line in modern medicine using the classical Greek concept of nemesis – retribution suffered by mortals when they strive to be god-like, following dreams unchecked by reasonable self-restraint. He linked this concept to modern medicine which he argued had reached a watershed in, amongst other things, producing clinical damage which outweighs its benefits; abrogating the rights of people to care for themselves; infringing privacy; alienating people by repressing pain and extending sick life; and turning us all into passive consumers as opposed to autonomous producers. This argument has its flaws – particularly given the rather backward-looking ethnocentric position that underlies it,^13^ the new advances in medicine that give greater hope of providing extended high quality lifespans,^14^ and the now higher level of user and carer participation in healthcare.^15^ However, the parallels with the fault line between standardization and personalization identified in delivering healthcare should now be evident. The tension highlighted in the editorial has been even more concretely represented on a longstanding and pertinent basis, though, in the case of CAM, to which we now turn as it amplifies a number of strands of the argument presented.

## Amplifying Arguments: The Case of Complementary and Alternative Medicine

In Western healthcare, CAM is defined in terms of practices that lie outside orthodoxy established by the dominant medical profession and based on biomedicine. As such, it covers a very diverse cluster of therapies – ranging from acupuncture and aromatherapy to herbalism and homeopathy.^16^ These therapies do not share everything in common, with distinctions including both the nature of the specific practices and the underpinning philosophies adopted. However, what tends to bind CAM practitioners together – at least those operating outside of medical orthodoxy – is that most are committed to a holistic approach based on treating the ‘whole person.’^17^ This concept, which has resonance with the customized approach highlighted in the editorial, has a number of meanings, including forging a philosophical link between mind and body. Typically in this context, though, it encompasses a labour-intensive orientation to personalizing healthcare. There is also usually an interlinked subscription to assessing effectiveness on the basis of qualitative individual case studies and the subjective views of users about value. As such, most CAM therapies have roots going back not just to traditional folk medicine, but also to bedside medicine in the eighteenth century when diagnosis and treatment by physicians was centred on a genuine dialogue between doctor and patient – before this ground was usurped through first the rise of hospital medicine and then laboratory medicine.^18^

As noted in the editorial, the pendulum has swung in recent times towards a greater degree of engagement of the patient in orthodox medical practice. However, despite a desire by practitioners to retain their independence through professionally policed boundaries in an increasingly specialized domain, medicine remains heavily weighted towards standardization centred on commonality of practice enforced through a range of professional regulatory mechanisms, ranging from the formally prescribed educational curriculum to the informal practices of the editorial gatekeepers of mainstream medical journals.^19^ In all this, biomedical principles tend to prevail – in medicine as well as the allied health professions – frequently based on the results of randomized controlled trials on which EBM is centred. Associated assessments of effectiveness tend to be quantitative and objectified, with clear restrictions on patient empowerment imposed by the scientific paradigm of biomedicine.^20^ Indeed, even groups like doctors, nurses and physiotherapists who have taken up CAM in the Anglo-American and other contexts have tended to link these to orthodox explanations of their operation and sought to use small-scale matched experimental and control group methodology to underwrite the therapies they employ. So too have the handful of CAM therapists, such as chiropractors and osteopaths who have managed to professionalize by gaining statutory monopolies over their practice.^21^ The largely customized case of CAM then is salutary in encompassing the very explicit tensions with the more standardized orthodox biomedical approach.

## Conclusion: Future Actions

The case of CAM therefore serves to amplify and further illustrate in a stark way the divide between the institutional logics of customization and standardization as outlined in the editorial – not least in the context of the most recent attack by orthodox Western scientists on CAM, which has added to its marginalization.^22^ In addition, it accentuates that this split in logics is not just reflected in a division between managers and health professionals, but also helps to define – ideologically at least – the frequent gulf between medical orthodoxy and CAM, as well as the *modus operandi* of both types of practitioners. Having said this, we should be very grateful for the editorial by Manning and Exworthy since – whatever its weaknesses – it has drawn attention to an increasingly recognized and important fissure in contemporary healthcare, albeit one that requires much more research. In terms of future actions, the challenge that lies ahead therefore is to communicate its existence as widely as possible to enhance debate. The aim should be to promote more finely textured work in healthcare on the tensions between these logics at local, regional, national and international levels. It is to be hoped that this will not only be of great interest to academics, but also lead to understandings that can be applied to future health policy to the benefit of users of services in modern neo-liberal societies.

## Ethical issues

Not applicable.

## Competing interests

Author declares that he has no competing interests.

## Author’s contribution

MS is the single author of the paper.
